# Clinical and Paraclinical Characteristics of Non-Classic Phenylketonuria

**DOI:** 10.22037/ijcn.v15i3.30519

**Published:** 2021

**Authors:** Marjan SHAKIBA, Hedyeh SANEIFARD, Mohammad Reza ALAEI, Asieh MOSALLANEJAD, Mojtaba LOTFI, Mehrdad YASAEI, Elahe ALIZADE NADERI

**Affiliations:** 1 Pediatric Endocrinology and Metabolism , Mofid Children’s Hospital, Shahid Beheshti Unversity of Medical Sciences, Tehran, Iran; 2 Pediatric Resident Endocrinology and Metabolism, Mofid Children’s Hospital, Shahid Beheshti Unversity of Medical Sciences, Tehran, Iran

**Keywords:** Phenylketonuria, Non-classic PKU, Hyperphenylalaninemia, Hypotonia, Neoptrin

## Abstract

**Objective:**

Phenylketonuria (PKU) is one of the most common inherited metabolic diseases, which is classified into classic and non-classic types. It is estimated that 2% of children with PKU develop a severe and progressive neurological disease, called non-classic (malignant) PKU. This study aimed to demonstrate the clinical features, laboratory findings, and diagnostic/therapeutic characteristics of non-classic PKU patients referred to a tertiary referral center for children in Tehran, Iran.

**Materials & Methods:**

In this study, background information, such as gender and age, clinical manifestations, laboratory findings, and response rate to conventional treatment, was investigated in patients with non-classic PKU, who were referred to Mofid Children’s Hospital in Tehran, Iran, through neonatal screening.

**Results:**

Twenty patients with a diagnosis of non-classic PKU were included in this study. The mean age of the patients was 6.00±2.81 years (range: 2-12 years), and 45.0% were male. In patients with a late diagnosis, the most common presentations were motor developmental delay (15.0%), skin and cutaneous manifestations (15.0%), seizure (5.0%), and restlessness (5.0%). The overall response rate to treatment was 85.0%. Factors that predict good response to treatment included female gender, higher neopterin level, and lower age at diagnosis and management.

**Conclusion:**

In conclusion, about half of patients with non-classic PKU remain asymptomatic, which is due to early diagnosis via neonatal screening. Also, higher age at diagnosis and treatment, besides low neopterin levels, may be useful as prognostic factors.

## Introduction

Inborn errors of metabolism are rare genetic disorders in which the body cannot turn food into energy. Although all of these disorders are rare, their number and diversity are high; therefore, they make up the most common group of genetic disorders. Untreated metabolic disorders, based on their pathophysiology, may result in severe presentations, such as mental retardation (1). Phenylketonuria (PKU) is one of the most common inherited metabolic diseases in the world, which is commonly classified as classic (phenylalanine hydroxylase [PAH] deficiency) and non-classic (severe and malignant tetrahydrobiopterin [BH4] deficiency) (2-4).

PAH deficiency is an inherited autosomal recessive defect in the metabolism of phenylalanine, causing a significant increase in phenylalanine to levels above 20 mg/dL (1200 µmol/L); this type of PAH deficiency is called classic PKU. A small percentage of patients have no PAH deficiency but lack BH4 synthesis and recycling as a cofactor of PAH. This leads to a defect in the biosynthesis of catechol amines and serotonin, resulting in a more serious neurological complication, called non-classic (malignant) PKU (5).

Generally, inborn errors of metabolism comprise a heterogeneous group of disorders, caused by mutations in one of the genes encoding enzymes involved in biosynthesis (guanosine triphosphate cyclohydrolase [GTPCH] or 6-pyruvoyl-tetrahydropterin synthase [PTPS]) or regeneration (pyruvate carboxylase deficiency [PCD] or dihydropteridine reductase [DHPR] of BH4. Hyperphenylalaninemia (HPA), which is present in the majority of patients, may be detected through neonatal PKU screening programs. Diagnosis of non-classic PKU among patients with HPA requires the measurement of pterins (neopterin and biopterin) in the urine, DHPR activity in the blood, and neurotransmitter metabolites in the cerebrospinal fluid (CSF).

The primary enzyme defect and its severity, the outcome of BH_4_ challenge, type of mutation, and response to therapy are some of the criteria used to define the prognosis of PKU. The terms severe/general and mild/peripheral/partial should be used, according to the patient’s need for treatment with neurotransmitter precursors (5, 6). Use of older terms, such as atypical PKU (non-PKU HPA) or malignant PKU, should be avoided, as in most cases, a BH_4_ deficiency is no longer lethal (6). Today, older children are detected based on different clinical symptoms, such as hypotonia of the trunk, hypertonia of the extremities, and often myoclonic seizures, unresponsive to a low-phenylalanine diet. Neonates with PKU are asymptomatic at the beginning of the disease and may not show relevant abnormalities within the first few months; therefore, a timely diagnosis can prevent these damaging processes (7).

Despite the high prevalence of PKU and the differences observed in the epidemiological and clinical aspects of this disease, studies on non-classic PKU are limited. In this study, we aimed to evaluate the clinical features, laboratory findings, and diagnostic and therapeutic properties of non-classic PKU patients, referred to a tertiary referral center for children in Tehran, Iran.

## Materials & Methods

In this cross-sectional study, the clinical and laboratory information of patients with non-classic PKU was collected by reviewing their hospital records. Their background information, such as gender, age, clinical manifestations, laboratory findings, and response to conventional treatment, was also investigated. 

Descriptive analyses, including mean±standard deviation (SD) for quantitative variables and frequency (percentage) for comparative variables, were used to delineate the data. Chi-square test, t-test, or Mann-Whitney U test was used for comparison of variables. For statistical analysis, SPSS version 22.0 (released in 2013, IBM Corp., Armonk, NY, USA) was used. P-values less than 0.05 were considered statistically significant. 

Patients with incomplete medical records or without follow-up were excluded from the study. Informed consent was obtained from all human adult participants and the parents or legal guardians of minors. The Research Ethics Committee of Shahid Beheshti University of Medical Sciences approved the study protocol. 

## Results

Twenty patients with non-classic PKU were included in this study. The mean age of the patients was 6.00±2.81 years (range: 2-12 years), and 45.0% were male. The baseline information of the patients is summarized in Table 1. A family history of PKU was only reported in 15.0% of the patients, while consanguinity was found in 70.0%. 

Overall, 30% of the patients in this study were diagnosed in the national screening program. In the remaining patients with a late diagnosis, the most common presentations were motor developmental delay (15.0%), skin and cutaneous manifestations (15.0%), seizure (5.0%), and restlessness (5.0%). None of the patients had organomegaly or cardiopulmonary abnormalities. The mean age at diagnosis was 37.06±31.67 months. The most common subtype was DHPR deficiency (45.0%), followed by PTPS deficiency (30.0%) and PCD (25.0%). 

An appropriate response to treatment was found in 17 out of 20 patients, yielding an overall response rate of 85.0%. Positive response to treatment was significantly higher in females than in males (100% vs. 66.7%; P=0.038), and was independent of family history of PKU (P=0.998), parental consanguinity (P=0.521), and different subtypes of PKU (P=0.277). As shown in Table 2, the mean neopterin level was significantly lower in patients with treatment failure as compared to those who responded appropriately to treatment (2.10±0.52 vs. 11.52±8.86; P<0.001). However, response to treatment was not dependent on the level of biopterin or phenylalanine. Besides, those with a higher chance of better response to treatment had a lower mean age at diagnosis and upon treatment (Table 2). Therefore, the main indicators for predicting a better treatment response were female gender, higher neopterin levels, and lower age at diagnosis and management. 

**Figure 1:**
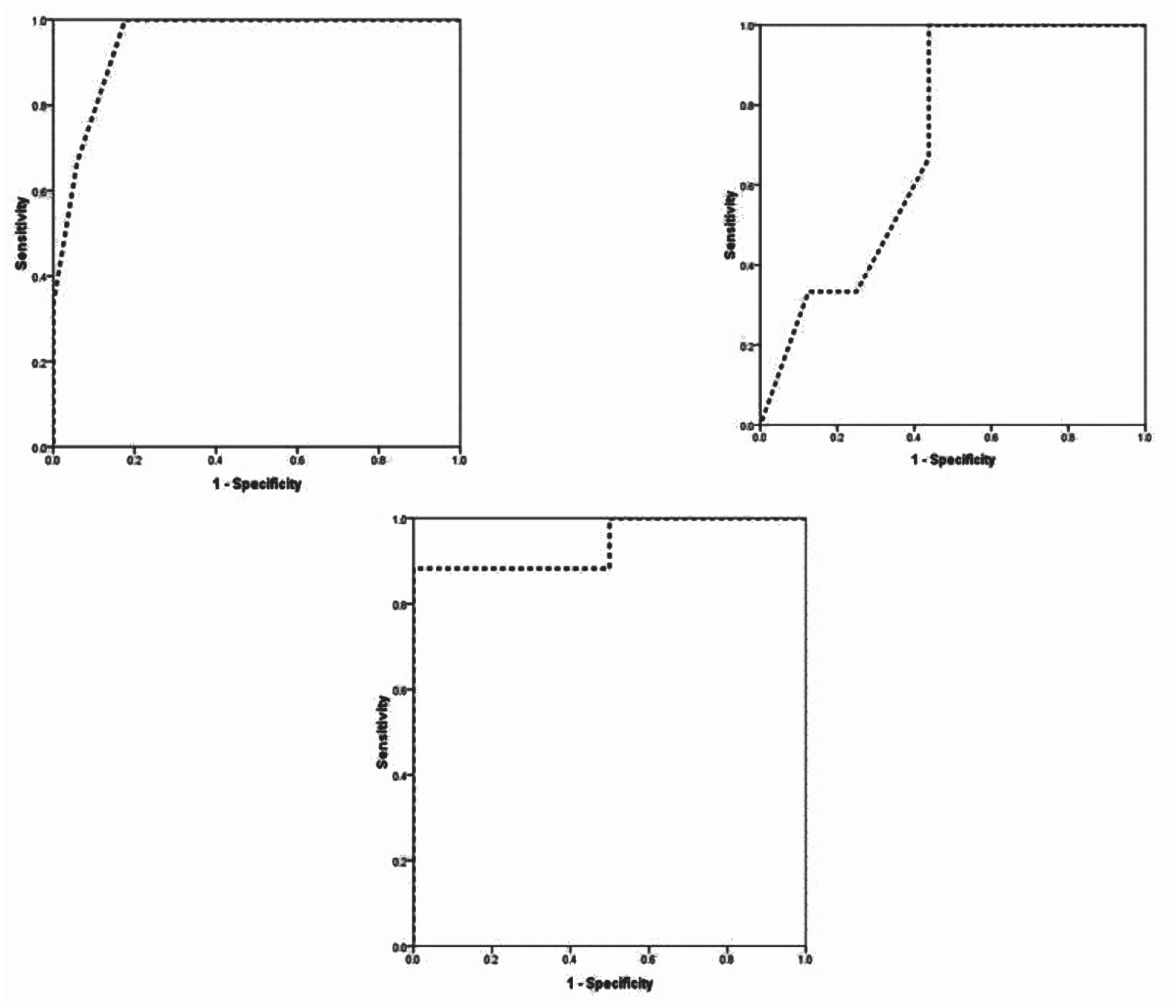
The ROC curve analysis to predict successful PKU treatment based on the age at diagnosis, patient's age and the level of neoptrin

Based on the receiver operating characteristic (ROC) curve analysis, higher age (AUC=0.948), higher age at diagnosis (AUC=0.719), and neopterin deficiency (AUC=0.938) could effectively predict poor prognosis in non-classic PKU patients. The best cut-off values of variables for predicting better outcomes were 6.5 years for the patient's age (sensitivity of 100% and specificity of 81.2%), 36 months for age at diagnosis (sensitivity of 100% and specificity of 56.2%), and 3.50 years for the neopterin level (sensitivity of 87.5% and specificity of 100%) (Figure 1).

**Table 1:** Baseline characteristics of study population

Mean age, year	6.00 ± 2.81
Male gender	9 (45.0)
Mean weight, kg	2.65 ± 0.53
Mean height, cm	47.75 ± 3.02
Mean head circumference, cm	33.50 ± 1.97
Family history of PKU	3 (15.0)
Parents relative condition	14 (70.0)
Clinical manifestations	
Restlessness	1 (5.0)
Skin problems	3 (15.0)
Motor delay	3 (15.0)
Thalassemia minor	2 (10.0)
Seizure	1 (5.0)
Mean age at diagnosis, month	37.06 ± 31.67
Mean level of neoptrin	10.53 ± 8.86
Mean level of bioptrin	2.31 ± 2.13
Mean level of phenyl alanine	9.83 ± 4.17
Types of non-classic PKU	
DHPR deficiency	6 (45.0)
PCD deficiency	5 (25.0)
PTPS deficiency	6 (30.0)
Response to treatment	
Successful	17 (85.0)
Failed	3 (25.0)

**Table 2:** Response rate according to baseline indicators

Item	Response (+)	Response (-)	P value
Mean age	5.29 ± 2.23	10.00 ± 2.65	0.004
Mean weight	2.66 ± 0.56	2.60 ± 0.36	0.855
Mean height	48.24 ± 2.41	45.00 ± 5.19	0.394
Mean head circumference	33.54 ± 2.11	33.33 ± 1.16	0.875
Mean age at diagnosis	36.38 ± 32.56	64.00 ± 18.33	0.025
Mean level of neoptrin	11.52 ± 8.86	2.10 ± 0.52	0.001
Mean level of bioptrin	2.32 ± 2.45	2.31 ± 3.18	0.996
Mean level of phenyl alanine	8.76 ± 2.56	10.90 ± 5.79	0.218

## Discussion

In the present study, the clinical features, laboratory findings, and response to treatment were investigated among non-classic PKU patients. Also, factors associated with a better prognosis were examined in these patients. In the current study, the effect of female and male gender was similar. Most of the patients had a negative family history of the disease, while consanguinity was found in 70% of the patients. The common clinical manifestations included motor delay, eczema, seizure, and restlessness; nevertheless, a significant proportion of patients (11 out of 20) were completely asymptomatic. Organomegaly was not detected in any of the patients; all of them showed normal cardiopulmonary responses. The average time from birth until diagnosis was about three years, which is a significant time interval. Besides, the response rate to treatment was 70% in our patients.

In this regard, poor prognosis has been reported in children with higher age at diagnosis, older age, and low neopterin levels; these factors predicted the treatment failure in the patients. Based on the epidemiological distribution of PKU in Iran, the results may differ from those reported in other parts of the country. In a study by Koochmeshgi et al., the parents were first cousins in 56% of cases (8). Moreover, in a study by Eshraghi et al. in Mazandaran, Iran, 70% of the patients were boys, and the mean age of diagnosis was 20 months. Also, in their study, 60% of the patients had consanguineous parents, and 10% had a history of PKU in their siblings (9). Besides, Morovatdar et al. reported mental retardation in 60% of patients and seizure in 36% of patients with PKU in Mashhad, Iran. In 80% of patients, consanguineous parents were reported, and 49% of the patients were male (10). Therefore, it can be concluded that the epidemiological distribution of PKU in different regions of the country is not completely homogeneous.

In a case-series of seven malignant PKU cases in Ireland, two patients had PTPS deficiency, and five patients showed DHPR deficiency. This finding is similar to our results, which revealed that DHPR deficiency (45.0%) was the most common subtype, followed by PTPS deficiency (30.0%) (11). Based on the evaluation of response to treatment and its dependence on indicators, such as age, age of diagnosis, and neopterin level, our findings are consistent with a study by Dayasiri et al., which reported a case of progressive neurological dysfunction secondary to malignant HPA, with a markedly reduced neopterin level and an undetectable biopterin level (12).

Some researchers have confirmed treatment failure in older patients or those with a late diagnosis. It is well-documented that diagnostic measures should be taken before the third week of life, and treatment must start before the first month of diagnosis (13). It has also been shown that the effect of treatment on intellectual status decreases with age. Therefore, to prevent the complications of the disease, the onset of treatment after 12 to 14 years will not exert significant effects on the patient’s mental status (14, 15). Based on the current guidelines, treatment should begin within the first ten days after birth; this will be only possible when diagnosis and treatment are done in the initial screening of the disease. Otherwise, with delayed diagnosis and treatment, irreversible complications, such as delayed disease development, recurrent seizures, and significant mental retardation, may occur. According to our findings, factors, such as age at diagnosis >3 years and a neopterin level <3.50 can be considered as helpful clinical criteria for a poor prognosis.

In the present study, there were some potential limitations. First, because of the rarity of PKU, we had a small sample size, which made it impossible to evaluate and remove the confounding factors for determining the predictors of prognosis. Second, it was not possible to evaluate the genetic variations in different PKU subgroups. Third, due to the cross-sectional design of this study, it was not possible to investigate the patients’ response to long-term treatment or their long-term survival.

## In Conclusion

According to the findings of this study, lower age at diagnosis and treatment and a high neopterin level are useful prognostic factors in non-classic PKU.
